# Copper Delivery to Chloroplast Proteins and its Regulation

**DOI:** 10.3389/fpls.2015.01250

**Published:** 2016-01-12

**Authors:** Guadalupe Aguirre, Marinus Pilon

**Affiliations:** Biology Department, Colorado State UniversityFort Collins, CO, USA

**Keywords:** plastocyanin, photosynthesis, copper deficiency, copper transporting P-type ATPase, polyphenol oxidase, superoxide dismutase, Cu-miRNA

## Abstract

Copper is required for photosynthesis in chloroplasts of plants because it is a cofactor of plastocyanin, an essential electron carrier in the thylakoid lumen. Other chloroplast copper proteins are copper/zinc superoxide dismutase and polyphenol oxidase, but these proteins seem to be dispensable under conditions of low copper supply when transcripts for these proteins undergo microRNA-mediated down regulation. Two ATP-driven copper transporters function in tandem to deliver copper to chloroplast compartments. This review seeks to summarize the mechanisms of copper delivery to chloroplast proteins and its regulation. We also delineate some of the unanswered questions that still remain in this field.

## Introduction

It is estimated that at least a third of all known proteins require a metal cofactor (Waldron et al., 2009). Copper (Cu) is essential for most living organisms including higher plants. In living cells Cu is predominantly found in two redox states, Cu^2+^ (cupric) and Cu^+^ (cuprous). The utility of Cu is related to its capacity to switch redox states in the cellular environment (i.e., become oxidized and reduced while bound to a protein) and the majority of Cu proteins (∼90%) found in nature function as oxidoreductases (Waldron et al., 2009). In green tissue of plants a large fraction of the Cu is present within the chloroplasts, corresponding to roughly a third of the Cu in leaves of soil-grown, well-fertilized, *Arabidopsis thaliana* (Shikanai et al., 2003). The fraction of Cu in a leaf that is allocated to plastids probably varies with Cu supply, however, (Burkhead et al., 2009). In this review we aim to describe the mechanism of Cu delivery to chloroplast proteins and its regulation. Emphasis is given on new models and outstanding questions.

## Chloroplast Copper Proteins

**FIGURE 1:**
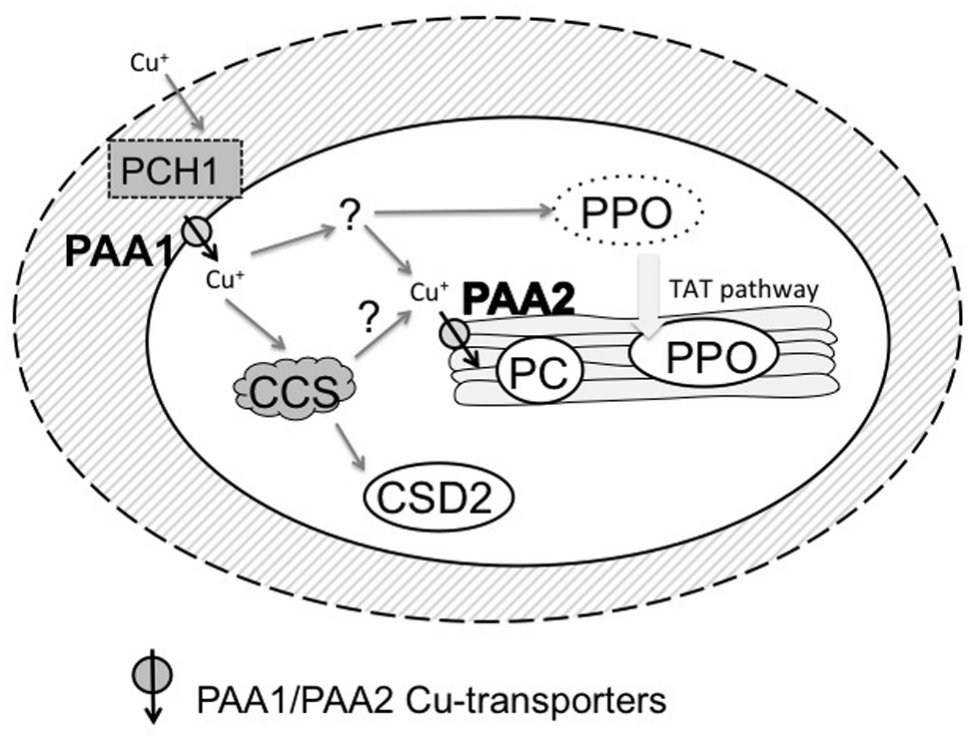
**Chloroplast copper proteins and delivery systems.** CSD2 (copper/zinc superoxide dismutase) is active in the stroma and receives Cu ions from CCS (the copper chaperone for superoxide dismutase). PC is active in the thylakoid lumen. PPO is found in the thylakoid lumen but may receive its Cu ion in the stroma before entering the thylakoid space via the TAT protein translocation pathway. PAA1 is a copper transporter in the envelope required to supply Cu ions to CSD2, PC and presumably also PPO. PAA1 receives Cu ions from the plastid copper chaperone 1 (PCH1). Cu ions may reach PCH1 in the inter membrane space of the envelope via diffusion through porins in the outer membrane, perhaps bound to low molecular weight chelators such as glutathione. Alternatively, PCH1 might pick up Cu ions in the cytosol and diffuse in via pores in the outer membrane. PAA2 is a homologs copper transporter in the thylakoid membrane required to deliver Cu ions to PC. A protein donating Cu ions to PAA2 has not been identified yet.

In chloroplasts, three major Cu proteins have been described (see **Figure 1**). In the stromal compartment, an isoform of copper/zinc superoxide dismutase is active. The enzyme is encoded by the *CSD2* gene in *Arabidopsis thaliana* (Kliebenstein et al., 1998). CSD2 maturation has an absolute requirement for a conserved CCS called CCS (Abdel-Ghany et al., 2005b; Chu et al., 2005; Huang et al., 2012) which functions to deliver and insert Cu into this enzyme. In contrast, the major cytosolic isoform of copper/zinc superoxide dismutase (CSD1 in *Arabidopsis*) can mature without CCS, albeit with low efficiency (Huang et al., 2012). Although copper/zinc superoxide dismutases are highly conserved in plants and other eukaryotes, their biological significance in plants has been elusive. A report of strong phenotypes for a loss of function (knock-down) mutant of CSD2 (Rizhsky et al., 2003) was in retrospect based on a insertion mutant that upon further study did not seem to affect CSD2 protein expression at all (Cohu et al., 2009). Phenotypes of CCS loss-of function mutants, which have no detectable CSD2 activity and very low CSD1 activity, are very mild when compared to the wild-type (Dugas and Bartel, 2008; Cohu et al., 2009) or require extreme stress to become noticeable (Sunkar et al., 2006). Redundancy with chloroplastic FeSOD may be one factor affecting Cu/ZnSOD loss of function phenotypes (Cohu et al., 2009). The green alga *Chlamydomonas reinhardtii* does not encode for Cu/ZnSOD but it contains FeSOD in the chloroplast.

Plastocyanin is a small Cu protein in the thylakoid lumen and serves as an electron carrier between the cytochrome-*b_6_f* complex and PSI in plants and is essential for photo-autotrophic growth in plants (Weigel et al., 2003). In contrast, similar to some cyanobacteria, *Chlamydomonas* can use the heme-containing cytochrome-c_6_ when Cu becomes limiting, as an alternative to PC (Kropat et al., 2015). In most plant genomes including *Arabidopsis* and poplar two PC isoforms are encoded. The two isoforms have essentially equivalent function regarding electron transport activity but in *Arabidopsis* one protein isoform (PC1) accumulates at a lower level whereas a second isoform (PC2) is much more abundant but also more sensitive to Cu availability at the protein level (Abdel-Ghany, 2009; Pesaresi et al., 2009). In poplar, both PC isoforms seem to be expressed at a comparable level (Ravet et al., 2011). PC was the first plant Cu enzyme that was cloned and its biogenesis is very well studied. The PC precursor has a bipartite N-terminal targeting sequence consisting of first a transit peptide, which functions to bring the protein into the stroma, followed by a signal sequence that serves for interaction with the ATP-dependent SEC machinery for translocation to the thylakoid lumen (Smeekens et al., 1986). Because protein transport over the envelope and thylakoid membranes via the SEC pathway requires unfolded polypeptide substrates, Cu must insert into apo-PC in the thylakoid lumen (for a review on chloroplast protein transport see Jarvis, 2008).

A third abundant Cu enzyme is PPO, also known as tyrosinase, which is present in the thylakoid lumen of many plants including poplar and spinach where it was discovered as the first Cu enzyme in plants (Arnon, 1949). However, PPO is absent from *Arabidopsis* (Schubert et al., 2002) and *Chlamydomonas*. The PPO enzymes contain 2 Cu atoms per monomer. *In vitro*, PPO can catalyze the formation of ortho-diphenols or ortho quinones from mono-phenols or ortho-dihydroxyphenols (See Mayer, 2006). The PPO substrates are not found in the thylakoid lumen, but are stocked in other cellular compartments such as vacuoles, and cell disruption by herbivory would allow PPO to form the dark-brown colored products in disrupted cells that make plant tissue less digestible (Mayer, 2006). Thus, PPO is thought to function in defense to herbivory or pathogen attack (Constabel et al., 2000; Wang and Constabel, 2004). As is the case with PC, the PPO precursors contain a bipartite targeting sequence at their N-terminus, but in the case of PPOs the thylakoid transfer sequence contains the twin arginine sequence motif, which means that PPO should use the TAT system for thylakoid transfer (see Jarvis, 2008). Because the TAT system can translocate folded peptides this implies that PPO might acquire its two Cu atoms in the stroma.

## Principles of Copper Homeostasis

It helps to understand chloroplast Cu transport in the context of overall Cu homeostasis and the properties of Cu ions. Among biologically active metal ions, copper binds the most tightly to its ligands. Both cupric and cuprous ions bind tightly to S and N-containing ligands (Irving and Williams, 1948; Waldron et al., 2009). These ligands are abundant in the cytosol, either as parts of amino acid functional groups in proteins (particularly cysteine, methionine, and histidine) or in low molecular weight compounds such as glutathione (cysteine). Within the reducing environment of the cytosol, free thiols in proteins and glutathione are especially strong ligands for Cu^+^ ions (Waldron et al., 2009). Therefore, the cytosol will have a very high chelating capacity for Cu ions when compared to an extracellular or exoplasmic compartment (e.g., vacuole) and indeed only very low free Cu ion concentrations are found inside cells (Rae et al., 1999). The very high cellular chelation capacity for Cu means that effectively a concentration gradient for free Cu ions should exist over the plasma membrane with a relatively low free Cu concentration in the cytosol, and this should help to provide a driving force for Cu ion uptake from the extracellular space. Conversely, export of Cu ions from the cytosol or a compartment with similar properties such as the chloroplast stroma should require metabolic energy. The strong tendency of Cu to bind to intracellular sites also makes it possible for Cu ions to replace other metals, causing toxicity. For this reason and in order to ensure correct delivery of Cu^+^, intracellular Cu-chaperones exist which scavenge Cu^+^ and deliver it to specific targets via protein-protein interactions. Finally, sensors that control Cu homeostasis should have a high affinity for the ion.

## Cellular Copper Transporters

Before turning to chloroplast-specific transport we want to first give a brief overview of cellular Cu transport and regulation because this affects the chloroplast also. Four classes of transporters have been implicated in Cu transport: COPT, ZIP, YSL, and HMA (see Williams et al., 2000; Burkhead et al., 2009).

### Roles of COPT, ZIP, and YSL Transporters

Copper enters eukaryotic cells by means of the CTR Cu transporters. The *Arabidopsis* genome contains six CTR-like sequences, which are called COPT1-6. COPT4 does not seem to be active (Sancenon et al., 2003). COPT 1, 2, and 6 are likely active at the cell surface (Sancenón et al., 2004; Jung et al., 2012), COPT3 may be active in an internal membrane and COPT5 is active in the tonoplast (Garcia-Molina et al., 2011; Klaumann et al., 2011). CTR/COPT transporters likely function as channels or carriers specific for Cu^+^ where the direction of transport is toward the cytoplasm (Eisses and Kaplan, 2005). The driving force for Cu^+^ uptake can be provided by both the strong capacity of the cytosol for Cu^+^ chelation as well as the typical orientation of the membrane potential, which is positively charged on the exoplasmic side due to proton exporting pumps. In the case of plant cells, reductases of the FRO family (Fro4/5) facilitate Cu uptake by reducing extracellular Cu^2+^ to Cu^+^, providing COPT proteins with a substrate (Bernal et al., 2012).

Whereas the role of COPT transporters is fairly well delineated, the picture is far less clear for the ZIP (Zrt and Irt like proteins; divalent metal transporters) and YSL (Yellow Stripe Like) transporter families. ZIP2 is clearly upregulated by Cu deficiency (Wintz et al., 2003; Bernal et al., 2012). ZIP2 and ZIP4 were reported to complement a yeast *ctr1* Cu uptake mutant (Wintz et al., 2003) but this result was not reproduced for ZIP2 in a more recent study, which instead indicated ZIP2 functions in Zn and Mn transport (Milner et al., 2013). The Yellow Stripe-Like (YSL) metal transporters, which were first identified in corn, are involved in the long-distance transport of metal-bound nicotianamine in *Arabidopsis* and rice (Waters et al., 2006; Chu et al., 2010; Zheng et al., 2012). For YSL family members YSL1, 2 and 3 roles in redistribution of Cu from leaves during senescence have been reported (Waters et al., 2006; Chu et al., 2010). YSL2 is strongly up-regulated in Cu deficiency at the transcript level in an SPL7-dependent process (Bernal et al., 2012). In excess-copper conditions, YSL1 and YSL3 proteins are down-regulated through sumoylation in *Arabidopsis* (Chen et al., 2011).

### HMA Transporters

**FIGURE 2:**
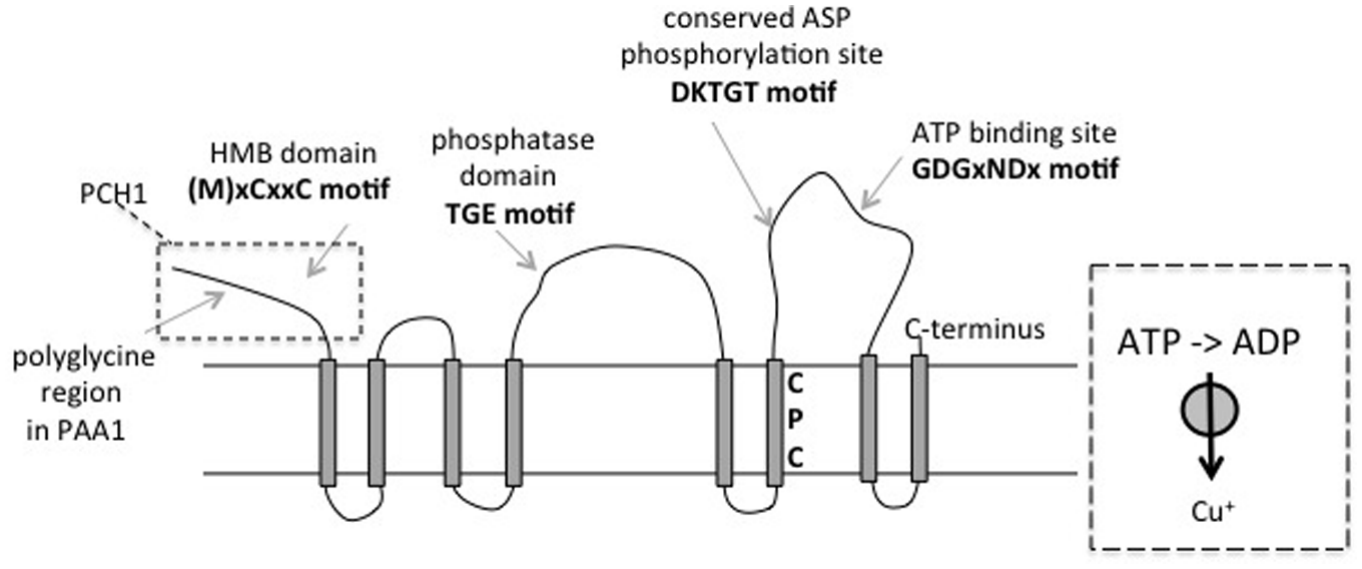
**Topology model of Cu+ transporting P-type ATPases such as PAA1 and PAA2.** The proteins have eight transmembrane segments. Major domains and sequence motifs are indicated. The region that corresponds to the copper chaperone PCH1, encoded in *Arabidopsis* by a PAA1 alternative splice form, is indicated in a box. The box on the right gives a simplified depiction of the protein in the same topology, which is used in **Figure 3** also.

An important class of Cu transporters is formed by a subclass of the metal transporting P1b-type ATPases or HMA proteins as they are called in plants (Williams et al., 2000; Hanikenne and Baurain, 2014). A schematic model for this type of transporter is given in **Figure 2**. Members of this family of transporters exist in two conformational states called E1 and E2. In all mechanistically studied P-type ATPases, hydrolysis of ATP after substrate binding is utilized to convert the E1 form of the enzyme to the E2 state in which a conserved ASP residue is phosphorylated. Subsequent release of the substrate on the *trans* side allows for phosphatase activity, which brings the enzyme back to the E1 state (for review see Rosenzweig and Argüello, 2012). In virtually all known P-type ATPases the ATP is bound on the cytoplasmic side and the substrates are exported from there (Rosenzweig and Argüello, 2012). The sequence motif CPC in TM region 6 is characteristic for Zn and Cu transporters (Hanikenne and Baurain, 2014). The specificity for Cu^+^ substrate is further determined by specific sequences in transmembrane regions 7 and 8 (Mandal et al., 2004). In other classes of P-type ATPases, the energy released by ATP hydrolysis can be used to pump ions such as H^+^, Ca^2+^, and Na^+^ against steep electrochemical gradients. In the cases of Zn and Cu it is evident that such large concentration gradients do not exist. However, the high chelating capacity of the cytosol for Zn and Cu ions presents a sizable thermodynamic barrier for export, which necessitates the utilization of metabolic energy for the transport of these ions from this compartment. Specific metallo chaperones are used to sequester the correct ion in the cytosol and deliver it to the cognate metal transporter via ligand exchange (Rosenzweig and Argüello, 2012). One of the best characterized Cu chaperones is ATX (anti-oxidant) from yeast, which has a ferredoxin-like fold and contains the consensus Cu binding motif MxCxxC. All Cu chaperones for P1B-type ATPases are similar and therefore this type of chaperone is called ATX-like (Pufahl et al., 1997). Most metal transporting P-type ATPases have one or more N-terminal heavy metal-binding (HMB) domain(s) which are also known as metal binding domains (MBD; (Hanikenne and Baurain, 2014). For Cu^+^ transporters these domains are structurally similar to ATX (Rosenzweig and Argüello, 2012). Work with bacterial Cu^+^ transporters has indicated that the HMB domain can accept metal from a ATX-like metallochaperone with a similar ferredoxin fold structure. Because the transporters maintain ATPase and transport activity without the HMB domain, and because this domain was shown to be inhibitory for ATPase activity in some cases, it can be proposed that the function of the HMB domain *in vivo* is perhaps regulatory (Rosenzweig and Argüello, 2012), but this has not been addressed in plants. The Cu chaperones that function with Cu-transporting ATPases bind via electrostatic interactions with a platform formed by a loop associated with transmembrane region 2 of the transporter and deliver sequentially two Cu^+^ ions directly to the transmembrane domains 6–8 that harbor the metal binding/transport sites (González-Guerrero and Argüello, 2008; Rosenzweig and Argüello, 2012).

Eight metal transporting P-Type ATPases are encoded in the *Arabidopsis* genome with homologs in most green eukaryotes (Williams et al., 2000; Hanikenne and Baurain, 2014). For four of the *Arabidopsis* HMA proteins a direct role in Cu homeostasis has been shown. HMA7 transports Cu from the cytoplasm to an endomembrane compartment where it can associate with the Cu binding site of the ethylene receptors (Hirayama et al., 1999). HMA5 in the plasma membrane removes Cu from the cell to allow xylem loading in the roots and prevent cellular Cu overload (Andres-Colas et al., 2006). HMA5 and 7 are likely to receive Cu from the ATX and or CCH cytosolic Cu chaperones for which interaction has been shown using yeast-two-hybrid assays (Puig et al., 2007). Two Cu transporting P-Type ATPases, PAA1/HMA6 and PAA2/HMA8 have been localized in plastids and these are described in more detail further below together with HMA1 a third ATP-driven metal transporter in the chloroplast.

## Copper Homeostasis

Because it affects chloroplast Cu transport pathways, it is necessary to briefly discuss cellular Cu homeostasis. When cellular Cu levels drop, a conserved transcription factor called SPL7 (Squamosa Promoter binding-Like 7) activates the transcription of a set of genes that together mediate a response to low Cu (Yamasaki et al., 2009). SPL7 in plants shares sequence similarity with *Chlamydomonas* CRR1 (the Copper Response Regulator) and activates transcription of genes that have a Cu Response element (CuRe) with the consensus sequence GTAC in it (Kropat et al., 2005; Yamasaki et al., 2009). SPL7 targets include several of the COPT transporters (COPT 1, 2, 6) and the potential cupric reductases FRO4 and 5 (Yamasaki et al., 2009; Bernal et al., 2012). Several highly conserved microRNAs, together called the Cu-microRNAs are also targets of SPL7 (Yamasaki et al., 2009; Bernal et al., 2012). These microRNAs in turn down-regulate the expression of Cu proteins including CSD2 and the PPOs (Yamasaki et al., 2007; Abdel-Ghany and Pilon, 2008; Cohu et al., 2009; Ravet et al., 2011). Since PC is not a target of a Cu-microRNA it can be hypothesized that the Cu-microRNAs serve to maintain a larger pool of Cu available for PC maturation, which is essential for photosynthesis (Burkhead et al., 2009). MiR398 targets CSD1, CSD2, and CCS as well as COX5b, a potential subunit of mitochondrial CCO but not a part of the Cu-binding core of this enzyme (Yamasaki et al., 2007). Mir397 and MiR408 target laccases and MiR408 also targets apoplastic plantacyanin (Abdel-Ghany and Pilon, 2008). MiR1444 targets PPO isoforms (Ravet et al., 2011). In *Chlamydomonas*, no miRNA regulation of Cu proteins exists but CRR1 regulates Cu economy by promoting the expression of cytochrome-*c_6_* and turnover of PC; further transcriptional regulation by CRR1 aims to further economize Cu use and optimize Fe acquisition in *Chlamydomonas* (Kropat et al., 2005, 2015).

## Copper Transport to Chloroplast Proteins

In the chloroplast two conserved Cu transporters have been identified (**Figure 1**). PAA1/HMA6 is localized in the chloroplast envelope (Shikanai et al., 2003; Catty et al., 2011). PAA2/HMA8 is localized in the thylakoids (Abdel-Ghany et al., 2005a). GFP fusions (Abdel-Ghany et al., 2005b), proteomic data (Catty et al., 2011; Tomizioli et al., 2014), and direct biochemical localization data, (Blaby-Haas et al., 2014) support the localization of these two proteins in *Arabidopsis*. In addition, PAA2/HMA8 was observed by immune EM in thylakoids in soybean (Bernal et al., 2007). Both PAA1 and PAA2 display all the characteristic motifs and domains expected for a Cu-transporting P-type ATPase (Abdel-Ghany et al., 2005a). A schematic structure is depicted in **Figure 2**. Phenotypes of *Arabidopsis* mutants indicated that both transporters are required for efficient maturation of PC, whereas only PAA1 is required for CSD2 activity. In addition, PAA1 mutants have lower Cu content in the entire chloroplast whereas PAA2 mutants have lowered Cu in the thylakoids (Abdel-Ghany et al., 2005a). Thus the two transporters function in tandem, as indicated in **Figure 1**. Homologs of PAA1 and PAA2 are present in eukaryotic green organisms including *Chlamydomonas* (Hanikenne and Baurain, 2014).

As discussed above, Cu-transporting P-type ATPases are expected to transport Cu away from the ATP binding site and to accept Cu from a metallo- chaperone. For PAA1 the topology in the inner envelope has been determined, which showed that the protein has its ATP binding site and thus also its substrate binding site on the intermembrane space side of the inner envelope membrane (Blaby-Haas et al., 2014). In *Arabidopsis*, two major splice forms are found for the PAA1 mRNA. One splice form, the full length mRNA, encodes for the full transporter. A second splice form is truncated and has an early stop codon, which leads in translation to a protein that only includes the transit peptide and HMB domain (Blaby-Haas et al., 2014). Since HMB domains are structurally very similar to Cu-chaperones it was proposed that this splice form encodes a chloroplast Cu chaperone called PCH1. PCH1 is conserved, in some plants it is formed from the alternative splice form of PAA1; in other plants gene duplication allowed a PAA1 sequence to evolve to encode the chaperone. Immunoblot analyses showed that the small Cu chaperone indeed is present in plants (Blaby-Haas et al., 2014). Direct biochemical measurements of ATPase activity for purified recombinant PAA1 and PAA2 showed that PCH1 can donate Cu to PAA1 but not to PAA2. Thus PCH1 seems to function in Cu delivery to PAA1 in the envelope intermembrane space of the chloroplast (Blaby-Haas et al., 2014). For PAA2 the topology is not determined directly. PAA2 functions to provide Cu to the lumen and thus we can expect PAA2 to have its ATP binding site in the stroma.

No regulation of transcript or protein levels has been observed in response to Cu availability for PAA1. For PAA2, however, regulation has been observed at the post-translational level. When *Arabidopsis* is grown on media with elevated Cu, much less PAA2 protein accumulates (Tapken et al., 2012). The mRNA of PAA2 is not affected by Cu feeding of plants, but instead Cu feeding mediates the turnover of the PAA2 protein via the conserved stromal CLP protease system (Tapken et al., 2015a). The observation that PAA2 is more stable in *paa1* mutants suggests that PAA2 turnover requires Cu in the chloroplast (Tapken et al., 2012). Interestingly, loss of pc2 resulted in increased PAA2 turnover Tapken et al. (2012). In a *paa1/pc2* double mutant, however, the regulation of PAA2 turnover was maintained similar to the wild type (Tapken et al., 2015b). Thus PC2 protein itself is not involved in PAA2 regulation. The CLP complex does not seem to be regulated by Cu (Tapken et al., 2015a). Rather, it can be proposed that PAA2 in association with Cu is more susceptible to turnover and this state is promoted by elevated Cu availability in the stroma or the absence of a Cu acceptor in the lumen.

It is not clear yet if there is a Cu chaperone for PAA2 in the stroma. *In vitro* experiments showed that CCS could interact with PAA2 and deliver Cu to stimulate ATPase activity of PAA2. The reported effect on the V_max_ of PAA2 for CCS bound to Cu was small in comparison to only Cu^+^ ions (Blaby-Haas et al., 2014). In addition, knock-out of CCS does not affect PC activity at all and CCS is also hardly expressed when Cu is below moderate availability due to miR398 regulation. Yet under these conditions PC still matures efficiently (Cohu et al., 2009). Because the CLP protease cleaves PAA2, it also cannot be excluded that the N-terminal HMB domain of PAA2 remains as a proteolytic fragment and this could be a chaperone for PAA2 in the stroma. But the free PAA2 HMB domain bound to Cu^+^ did not stimulate PAA2 ATPase activity at all and did not display Cu transfer activity (Blaby-Haas et al., 2014). Perhaps PAA2 receives Cu from a low molecular weight chelator such as glutathione in the stroma. Indeed ATPase activity is observed for PAA2 with only Cu^+^ present and in a phosphorylation assay PAA2 displayed a much higher affinity for free Cu compared to PAA1, which requires a chaperone (Blaby-Haas et al., 2014; Sautron et al., 2015).

## Effects of Copper-Micrornas on Cu Distribution in Chloroplasts

A potential miR408 target site is present in PAA2/HMA8 genes. However in *Arabidopsis* this site is not used (Bernal et al., 2012; Tapken et al., 2012). It seems likely that more indirect microRNA regulation via miR398 and miR1444 has important consequences for chloroplast Cu delivery to PC. To maintain growth and development of the photosynthetic apparatus during lower Cu availability, Cu delivery to PC should be prioritized. Induction of miR398 causes a drastic reduction in CCS and CSD2 protein expression whereas miR1444 will prevent PPO expression. This should allow remaining Cu in the stroma to be transported via PAA2 to PC (Burkhead et al., 2009). Could there be significance to the use of the TAT pathway by PPO? In agreement with its function in defense PPO is strongly induced by wounding. If PPO can pick up Cu in the stroma it would give plants a way to further prioritize Cu use in the emergency situation of biological attack. In such a case further maturation of PC is envisioned to be a lower priority.

## Outstanding Questions

The chloroplast Cu delivery system is now one of the best-understood trace metal delivery systems. However, several questions remain regarding the function of PAA1 and PAA2 as a system.

Firstly, alternative transport routes must exist for Cu in chloroplasts. All the tested *paa1* and *paa2* loss of function mutants are suppressed by elevated levels of Cu in the growth medium and suppression is more evident for *paa1* mutants (Abdel-Ghany et al., 2005a). Because both sequence analyses and immunoblot analyses have shown that all *paa1* alleles except for *paa1-3* and all *paa2* alleles are truly null mutants, the implication is that low affinity alternative Cu transport pathways must exist (Tapken et al., 2012; Blaby-Haas et al., 2014; Boutigny et al., 2014).

Another still puzzling observation is that *paa1/paa2* double mutants are seedling lethal even when given extra Cu in the media (Abdel-Ghany et al., 2005a). This observation is puzzling because synthetic lethality for two independent loci can be taken as support for functions of the encoded gene products in parallel pathways. Both *paa1* and *paa2* mutants are not lethal on soil, both are suppressed by Cu feeding and according to our model the two proteins function in sequence and not in parallel. Could we be wrong and could PAA1 and PAA2 function in parallel in the same membrane? This would require mis-targeting in the chloroplast of a fraction of the “normally” envelope-localized PAA1 to thylakoids or *vice versa* of PAA2 to the envelope. Such “mis-targeting” might explain both the suppression by Cu and the lethal phenotype of the double mutant. Given that PAA1 requires interaction with PCH1 and that PAA2 cannot accept Cu from PCH1 (Blaby-Haas et al., 2014) it seems unlikely that mis-targeted PAA1 or PAA2 could be functional. Similarly, modeling approaches have suggested that PAA2 but not PAA1 is suitable for Cu delivery to PC by direct docking between the transporter and apo-plastocyanin (Sautron et al., 2015). Rather, we favor the idea that loss of two high affinity transporters restricts Cu transport to the thylakoids so much that low affinity transport activities can no longer compensate (Abdel-Ghany et al., 2005a).

**FIGURE 3:**
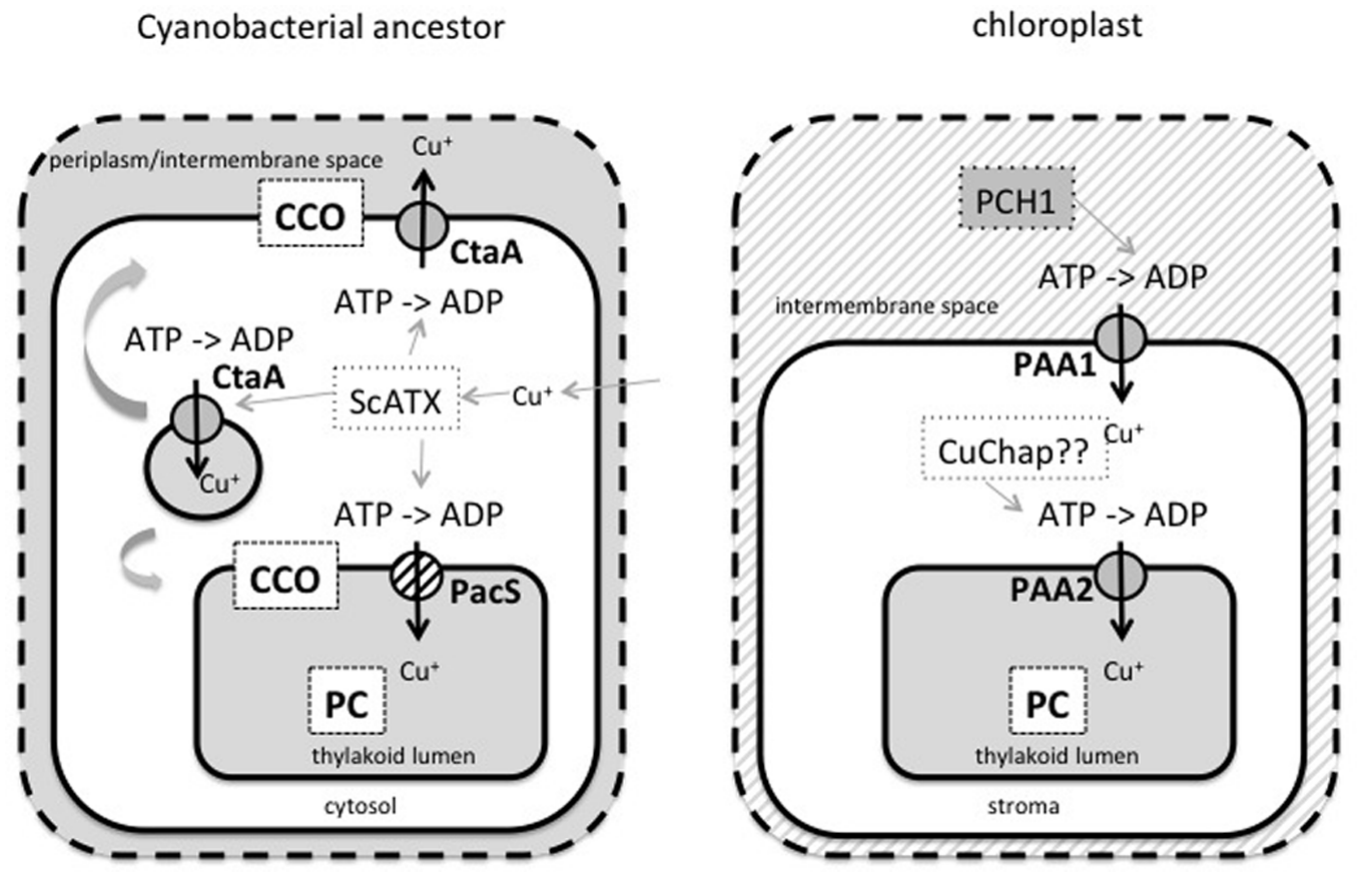
**Model for the evolution of chloroplast Cu transport from a hypothetical cyanobacterial ancestor of the chloroplast.** Cyanobacteria, such as *Synechocystis*, contain two major Cu proteins, PC in the thylakoids and CCO in both the thylakoids and inner membrane of the envelope. In these cyanobacteria two Cu-transporting P-type ATPases called CtaA and PacS are present together with a copper chaperone ScATX that can interact with both transporters. ScATX serves to sequester Cu ions that entered the cytosol by low affinity pathways and to deliver the Cu ions to CtaA and PacS. PacS is in the thylakoid membranes and loss of function affects both Cu tolerance and PC maturation. CtaA is presumably present in the inner membrane and maybe in addition in an internal vesicle. We propose that CtaA has the indicated topology, which allows the protein to deliver Cu to the exoplasmic side of membranes to allow maturation of both the CCO complex and PC. If CtaA is active in an internal vesicle then Cu ions can reach CCO and PC via a vesicular transport mechanism involving membrane fusion. After endosymbiosis gave rise to the chloroplast, both PacS and ScATX were lost. Nucleus encoded PAA1 and PAA2 then evolved from CtaA with the addition of a transit peptide for chloroplast targeting and for PAA1 also a poly-glycine stretch to ensure envelope retention and correct (novel) topology. In addition, alternative splicing or gene duplication allowed the evolution of the novel Cu chaperone PCH1 from PAA1/CtaA. In analogy to the use of the N-terminus of PAA1 as a copper chaperone, PAA2 may use its N-terminal region as a Cu chaperone in the stroma (CuChap, hypothetical at present) that might be derived from the full transporter via proteolytic processing. Alternatively PAA2 may receive Cu ions that bound to low molecular weight chelators in the stroma or from CCS protein (not shown).

How did PAA1 and PAA2 evolve? The chloroplast has evolved via endosymbiosis from a cyanobacterial ancestor in which Cu also needed to be supplied to PC and to CCO in the same membrane. In the cyanobacterium *Synechocystis PCC6803* two Cu-transporters, CtaA and PacS, and a Cu chaperone, ScAtx, have been described (Phung et al., 1994; Tottey et al., 2001, 2002). CtaA was thought to be located in the bacterial envelope inner membrane, ScAtx is cytosolic and PacS is in the thylakoid membrane (see **Figure 3**). It was proposed that CtaA functions as a Cu importer based on physiological measurements and Cu contents of mutant cells; and that PacS functions to transport Cu into the thylakoid lumen, which is important for PC maturation and the prevention of toxic effects of Cu in the bacterial cytosol (Tottey et al., 2001, 2002). Sequence comparisons show that both PAA1 and PAA2 are more similar to CtaA and thus PacS and ScAtx1 as well as COX seem to be lost from the chloroplast system after endosymbiosis (Abdel-Ghany et al., 2005a; Hanikenne and Baurain, 2014). The V_max_ kinetic parameter measured for both PAA1 and PAA2 is comparable to that of CtaA and about 10-fold lower than the V_max_ reported for PacS, which further supports the notion that PAA1 and PAA2 are more similar to CtaA (Blaby-Haas et al., 2014). Thus, it can be proposed that both PAA1 and PAA2 are derived from a CtaA-like cyanobacterial sequence. Which steps would have been required for the evolution of the chloroplast Cu-transporting P-type ATPases?

The scenario where CtaA functions as a Cu importer in the cyanobacterium (which is *not* as it is depicted by us in **Figure 3**) seems at first glance straightforward but there are, in our opinion, two issues with this model (Tottey et al., 2001, 2002). One is that CtaA in the cellular importer orientation should have a topology that is non-canonical from an evolutionary perspective in that it would have its ATP binding site not on the cytoplasmic side of the bacterial inner membrane but in the periplasm. Alternatively, CtaA could have its ATP binding site in the cytosol and import Cu toward its ATP binding site but in this case it would be the only Cu-ATPase that transports toward the cytoplasm and it would require a unique mechanism (this we consider a very unlikely scenario). Therefore we favor the idea that CtaA has a toplogy as indicated in **Figure 3**. The other issue associated with CtaA functioning as an importer would be that PAA2, if indeed derived from a CtaA-like sequence, would have flipped its topology in the membrane to now have its ATP binding site in the stroma of the chloroplast (equivalent to the bacterial cytosol). It is hard to envision a mechanism for this. Is there another, perhaps more parsimonious scenario for the evolution of PAA1 and PAA2 from a CtaA-like sequence? We propose the model in **Figure 3**. In this scenario CtaA is in an orientation in the cyanobacterium where it serves a biosynthetic function that allows it to donate Cu to exoplasmic (non-cytosolic) Cu enzymes such as PC and CCO. In the case of COX maturation, perhaps copper chaperones are utilized equivalent to the well-described factors that mediate COX maturation in mitochondria in the intermembrane space (Carr and Winge, 2003). CtaA in the topology proposed by us might be located in the inner membrane of the envelope. In addition, or alternatively, CtaA is in a hypothetical internal vesicle that would allow it to also facilitate Cu delivery to PC and COX in the thylakoids via vesicular transport. We believe that the model in **Figure 3** for CtaA function can be reconciled with all published data regarding phenotypes of cyanobacterial *ctaA* mutants and interactions with ScAtx (Tottey et al., 2001, 2002). For the evolution of PAA2 and PAA1 in chloroplasts we propose that they derive from a cyanobacterial CtaA protein perhaps present in an internal vesicle.

The model as depicted in **Figure 3** also has apparent problems, but we think that these issues can all be resolved. First, the model in **Figure 3** invokes that Cu enters the cyanobacterial cell via an alternative, not yet described, route. Low affinity yet somehow substantial Cu ion uptake by bacteria apparently does occur and is perhaps favored by the membrane potential and the high Cu chelating capacity of the cytosol relative to extracellular spaces. Indeed, many bacteria have extensive ATP-dependent Cu extrusion systems required to avoid Cu toxicity even if they lack specific Cu import pathways (Outten et al., 2001). Another possible weakness of the model as presented in **Figure 3** is that evidence for the types of vesicles that allow Cu trafficking is lacking in present day cyanobacteria. However, these types of vesicles would be by definition transient and we only need to invoke that an ancestor of the chloroplast utilized such vesicles. The final possible issue with the model now depicted in **Figure 3** is that PAA1 would have a flipped topology relative to CtaA. However, we postulate that the presence of the poly glycine domain of PAA1 serves as an envelope retention signal that at the same time forces insertion of the transporter in the correct new orientation. A poly glycine containing stretch was postulated to serve as an envelope retention signal for chloroplast TOC75 protein (Inoue and Keegstra, 2003). The poly glycine-containing stretch is a hallmark of PAA1 proteins and not found in PAA2 homologs (Abdel-Ghany et al., 2005a; Blaby-Haas et al., 2014; Hanikenne and Baurain, 2014).

Another question is whether there also is a pathway for the export of Cu from plastids? To regulate ion H^+^, Ca^2+^, and Na^+^ ion concentrations, cellular membrane systems are typically equipped with transport pathways for both directions. Indeed, in plants COPTs provide transport to the cytosol while HMA5 and 7 provide export. Is a similar scenario in place in the chloroplast? Is there a Cu exporter for thylakoids and the envelope membranes? In *Chlamydomonas* one additional function of PC next to electron transport might be that it serves as a Cu store upon which the cell can rely in times of impending deficiency (Kropat et al., 2015). In this state, CRR1 mediates expression of the Fe containing functional PC alternative cytochrome-*c_6_* while PC expression is turned off and PC turnover induced, perhaps via a Deg-type protease (Kropat et al., 2015). The Cu coming out of PC can then be utilized for other functions such as CCO in mitochondria and ferric reductase activities at the cell surface (Kropat et al., 2015). This scenario would rely on Cu export from the thylakoids and chloroplasts. Similarly, during senescence in higher plants Cu from plastids might be re-purposed for use in seeds or storage in stem parts. For *Arabidopsis* it was estimated that less than 30% of Cu going to seeds comes from senescing rosettes, which might imply that Cu re-routing from plastids is a minor pathway (Waters et al., 2006). What could be the Cu exporter? The P-type ATPase HMA1 is located in chloroplast envelope membranes (Seigneurin-Berny et al., 2006). It was originally proposed that HMA1 protein functions in Cu homeostasis based on phenotypic characterization of yeast expressing recombinant HMA1 (Seigneurin-Berny et al., 2006). Furthermore, a lower Cu content and reduced stimulation of ATPase activity by Cu ions for chloroplasts isolated from plants that lack HMA1 was observed, which led to the suggestion that HMA1 might function as an alternative for PAA1 in mediating Cu import into plastids (Seigneurin-Berny et al., 2006). However, a careful analysis of *paa1/hma1* double mutants suggests that HMA1 is unlikely to transport Cu into plastids since the double mutant is virtually identical to the single *paa1* mutant for the maturation of both PC and CSD2 (Boutigny et al., 2014). Overexpression of HMA1 in different backgrounds caused higher Cu levels to be observed in chloroplasts of the *paa1* mutant background but not in the wild-type background (Boutigny et al., 2014). We conclude that HMA1 affects Cu homeostasis somehow but the substrate (and direction of transport) for HMA1 remains unclear (see for a more extensive review and data on this topic see: Boutigny et al., 2014).

Another possible Cu export pathway might be formed by members of the YSL family, which was first described for its involvement in Fe acquisition. Recently two YSL members (YSL4 and 6) were described in the chloroplast envelope where they have a function in preventing Fe overload (Divol et al., 2013). Perhaps these proteins might also mediate some Cu export. Alternatively, Cu might be recycled from chloroplasts present in vacuoles following autophagy. Autophagy of plastids in lytic vacuoles might be an important mechanism to recycle precious micronutrients such as Cu and this field deserves more attention in the future (for a review see Blaby-Haas and Merchant, 2014).

Finally, a question is how Cu reaches the chloroplast surface, a question that is important to answer in view of the strong capacity of Cu to bind to proteins and thus to be sequestered in the cytosol (Burkhead et al., 2009). Perhaps cellular architecture should be considered here. Lysosomes, vacuoles and related compartments may play a role in providing Cu to the photosynthetic machinery. Under Zn deficiency, *Clamydomonas* sequesters Cu in a lysosome-related compartment, perhaps to prevent displacement of Zn with Cu in important Zn enzymes. This process is CRR1-dependent (Hong-Hermesdorf et al., 2014). It is not clear, however, if plants have a similar mechanism in place. The COPT5 transporter in the tonoplast membrane of plants allows Cu to re-enter the cytosolic Cu pool (Garcia-Molina et al., 2011; Klaumann et al., 2011). COPT5 is especially important during Cu starvation, a condition where it can be envisioned that it is beneficial to recycle precious Cu from organelles that are no longer needed, or damaged. In plant cells, the vacuole takes up a large volume and many organelles including chloroplasts are seen under the microscope to be pressed to the side of the cell by the vacuole. Under low Cu availability, close contact between the tonoplast and chloroplast envelopes would be an especially advantageous arrangement for Cu delivery to plastids. A very short distance between COPT5 and the chloroplast envelope surface would mean that Cu ions have to diffuse only a very small distance via outer membrane porins to reach PCH1 and PAA1 in the envelope and can virtually avoid the cytoplasm. It will be interesting to address these various questions in further research.

## Author Contributions

GA drafted the first manuscript. MP edited and finalized the MS.

## Conflict of Interest Statement

The authors declare that the research was conducted in the absence of any commercial or financial relationships that could be construed as a potential conflict of interest.

## Abbreviations

CCO: Cytochrome*-c* oxidase
CCS: Copper chaperone for SOD
CSD: Cu/ZnSOD or Cu/Zn-superoxide dismutase
HMA: Heavy Metal ATPase
PAA: P-type ATPase of *Arabodopsis*
PC: *Plastocyanin*
PPO: *polyphenol oxidase*
SPL7: Squamosa Promotor binding-Like 7
